# Novel mouse model of encephalocele: post-neurulation origin and relationship to open neural tube defects

**DOI:** 10.1242/dmm.040683

**Published:** 2019-11-14

**Authors:** Ana Rolo, Gabriel L. Galea, Dawn Savery, Nicholas D. E. Greene, Andrew J. Copp

**Affiliations:** Newlife Birth Defects Research Centre, UCL GOS Institute of Child Health, University College London, 30 Guilford Street, London WC1N 1EH, UK

**Keywords:** Brain, Malformations, Birth defects, Neural tube, Spina bifida, Skull

## Abstract

Encephalocele is a clinically important birth defect that can lead to severe disability in childhood and beyond. The embryonic and early fetal pathogenesis of encephalocele is poorly understood and, although usually classified as a ‘neural tube defect’, there is conflicting evidence on whether encephalocele results from defective neural tube closure or is a post-neurulation defect. It is also unclear whether encephalocele can result from the same causative factors as anencephaly and open spina bifida, or whether it is aetiologically distinct. This lack of information results largely from the scarce availability of animal models of encephalocele, particularly ones that resemble the commonest, nonsyndromic human defects. Here, we report a novel mouse model of occipito-parietal encephalocele, in which the small GTPase Rac1 is conditionally ablated in the (non-neural) surface ectoderm. Most mutant fetuses have open spina bifida, and some also exhibit exencephaly/anencephaly. However, a proportion of mutant fetuses exhibit brain herniation, affecting the occipito-parietal region and closely resembling encephalocele. The encephalocele phenotype does not result from defective neural tube closure, but rather from a later disruption of the surface ectoderm covering the already closed neural tube, allowing the brain to herniate. The neuroepithelium itself shows no downregulation of Rac1 and appears morphologically normal until late gestation. A large skull defect overlies the region of brain herniation. Our work provides a new genetic model of occipito-parietal encephalocele, particularly resembling nonsyndromic human cases. Although encephalocele has a different, later-arising pathogenesis than open neural tube defects, both can share the same genetic causation.

## Abstract (precis)

**Summary:** Encephalocele is a severe congenital brain defect that arises after neural tube closure, but can share a common genetic cause with defects of cranial and spinal neural tube closure.

## INTRODUCTION

Encephalocele is a severe birth defect of the skull and brain, with a median prevalence of 0.1-0.3 per 1000 births, but with considerable geographical variation in frequency (Zaganjor et al., 2016). The meninges, with or without brain tissue, herniate outside the skull, exposing the brain to potential damage both pre- and postnatally. Despite surgical repair soon after birth, later health problems are common, including hydrocephalus, epilepsy and learning difficulties. Encephaloceles emerge along the skull midline, with variation in rostro-caudal location, which can be fronto-ethmoidal, parietal, occipital or cervical. Generally, the prognosis worsens with posterior location, size of sac and increasing amount of herniated brain tissue (Kiymaz et al., 2010).

Although most cases are sporadic and of unknown causation, encephalocele can form part of a syndrome, as in trisomy 18, Knoblock syndrome (*COL18A1* mutation), amniotic band syndrome and warfarin embryopathy (Cohen and Lemire, 1982). Occipital encephalocele is best known as part of Meckel syndrome (MKS; overlapping with Joubert syndrome), in which individuals also exhibit polydactyly, polycystic kidneys and biliary defects. In recent years, mutations in several genes [e.g. *MKS1*, *MKS2* (*TMEM216*), *MKS3* (*TMEM67*), *CEP290*, *RPGRIP1L*] have been identified in various forms of MKS (Logan et al., 2010). Cellular analysis of the MKS-associated proteins has demonstrated a key role in the structure and function of primary cilia, and MKS is thus now classified as a ciliopathy.

Mice that incorporate mutations of some of the genes responsible for MKS display biliary, limb and kidney defects resembling the human syndrome, as well as defective ciliary structure and/or function (Cook et al., 2009; Goetz et al., 2017; Weatherbee et al., 2009). Although failure of cranial neural tube closure was described in a proportion of *Tmem67* (*MKS3*) null mice (Abdelhamed et al., 2013), none of the mouse models appear to exhibit herniation of brain tissue outside the skull, which would represent an encephalocele.

Although often classified as a ‘neural tube defect’ (NTD) (Logan et al., 2010; Rowland et al., 2006), the embryonic/fetal pathogenesis of encephalocele is less well understood than for other NTDs, particularly anencephaly and open spina bifida. The latter conditions result from defective closure of the neural tube (i.e. primary neurulation), as demonstrated by studies of NTD pathogenesis in mouse mutants (Copp et al., 2003). Of the many (>240) mouse mutants so far described, very few display a phenotype corresponding to encephalocele (Harris and Juriloff, 2010). Hence, the mouse data do not yet conclusively shed light on whether encephalocele is a primary neurulation defect or a post-neurulation anomaly, such as herniation of the closed neural tube through a skull defect.

Hence, progress in the field of encephalocele causation and early pathogenesis has been hampered by lack of a suitable animal model. Here, we describe a mouse model of encephalocele resulting from conditional deletion of Rac1, a small GTPase of the Rho family, in the non-neural (surface) ectoderm of the embryo and fetus. These mice exhibit open spina bifida (myelomeningocele equivalent) and, in some cases, exencephaly, the developmental forerunner of anencephaly (Camerer et al., 2010; Rolo et al., 2016, 2018). We show that a large proportion of these mice also develop occipito-parietal encephalocele, detectable from embryonic day (E) 13.5 onwards. The encephalocele displays a fully closed neural tube at the level of the lesion, with an associated skull defect. Hence, encephalocele is a post-neurulation anomaly, developmentally distinct from ‘open’ NTDs, and yet it can be caused by the same genetic defect as open spina bifida and exencephaly/anencephaly.

## RESULTS

### Generation of Rac1 conditional mutants and spinal neurulation phenotypes

*Rac1* was conditionally deleted by expressing Cre recombinase under control of the *Grhl3* promoter. Both *Grhl3^Cre/+^; Rac1^f/−^* and *Grhl3^Cre/+^; Rac1^f/f^* genotypes lack Rac1 expression mainly in the surface ectoderm (Rolo et al., 2016) and do not differ morphologically. Hence, these genotypes were pooled for analysis and denoted as Grhl3Cre-Rac1. They were compared with Cre-expressing control littermates *Grhl3^Cre/+^; Rac1^f/+^* and *Grhl3^Cre/+^; Rac1^+/−^*, which retain Rac1 expression, denoted as Grhl3Cre-Con. Littermates without Cre expression (*Grhl3^+/+^; Rac1^f/f^, ^f/+^, ^f/−^* or *^+/−^*) were denoted as Non-Cre controls (Table 1).

**Table 1.**
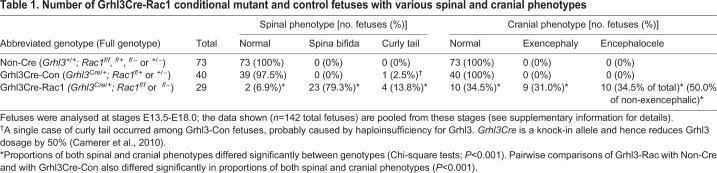
**Number of Grhl3Cre-Rac1 conditional mutant and control fetuses with various spinal and cranial phenotypes**

**Fig. 1.**
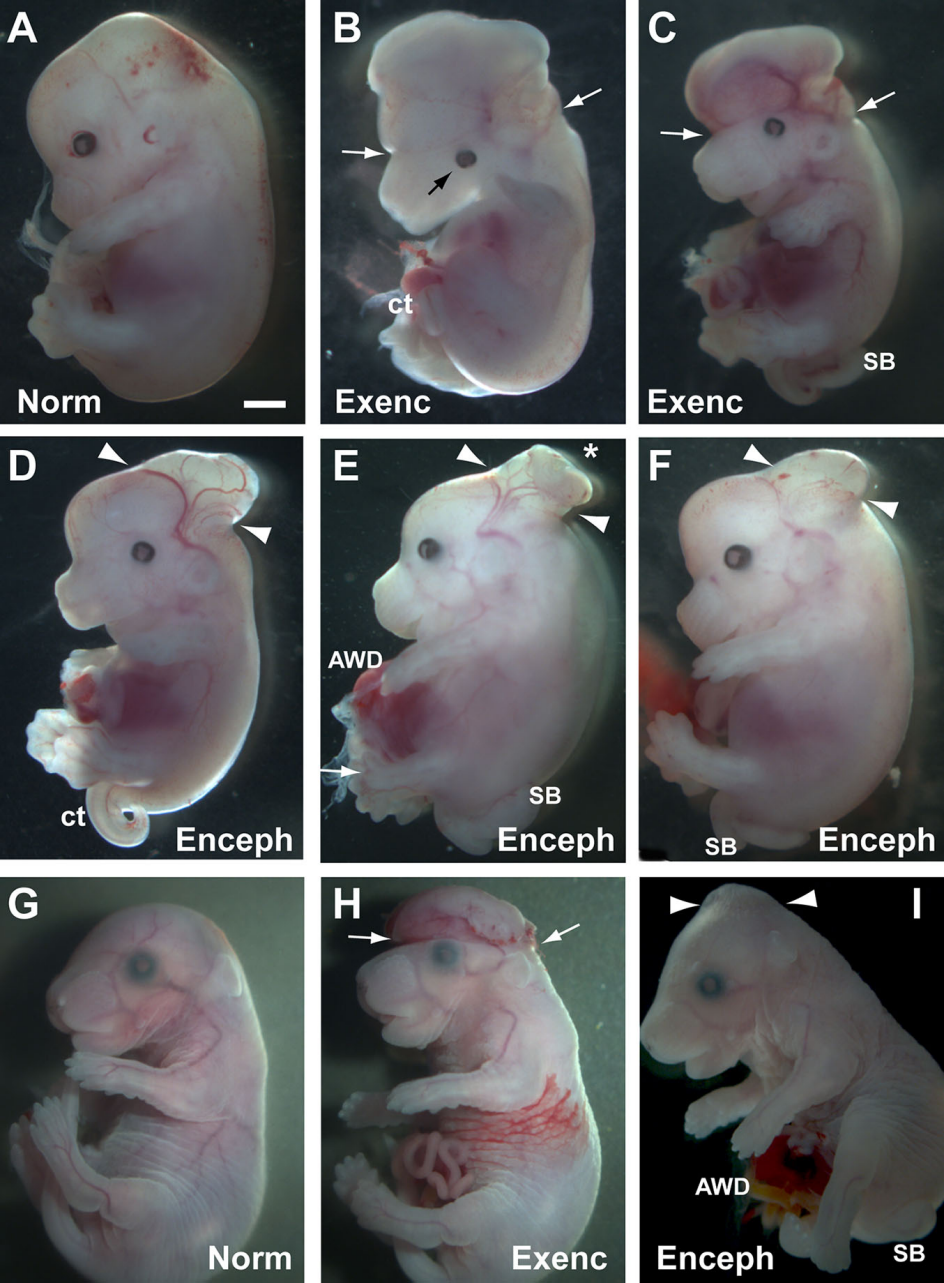
**Cranial and spinal defects in mouse fetuses with conditional deletion of Rac1.** (A-F) Non-Cre control (A) and Grhl3Cre-Rac1 mutant fetuses (B-F) at E13.5. Fetuses with exencephaly (Exenc) show the typical appearance of failed cranial neural tube closure with an extensive region of everted, open neural folds involving forebrain, midbrain and hindbrain (between white arrows in B,C). In contrast, three fetuses with encephalocele show a much more localised herniation of the occipito-parietal region (between arrowheads in D-F). In two cases, the herniation is smooth (D,F) whereas in the third there is a very localised area of open neural tube at the apex of the herniation (asterisk in E). All mutant fetuses shown have a spinal defect: spina bifida (SB in C,E,F) or curly tail (ct in B,D). Additionally, one fetus has an abdominal wall defect (AWD in E) with exteriorisation of the liver, and another has left-sided microphthalmia (black arrow in B). (G-I) Grhl3Cre-Rac1 mutant fetuses at E17.5. One mutant has no defects (G) whereas the others have exencephaly with normal spine (H) and encephalocele with spina bifida (I). The exencephalic tissue is partially collapsed on top of the head (compare H with B,C) and shows haemorrhage, indicative of progressive degeneration that ultimately leads to anencephaly. In contrast, the encephalocele retains a smooth nondegenerate appearance, similar to that observed at E13.5 (compare I with D-F). AWD is present in the fetus with encephalocele (I) whereas the exteriorised gut loops in the exencephalic fetus (H) were not considered AWD because the liver was not involved. The fetuses shown are representative of the phenotypes observed among the entire sample (*n*=142). See supplementary information for a list of all fetuses, showing those included in this figure. Scale bar in A represents 0.3 mm (A-F) and 1 mm (G-I).

Grhl3Cre-Rac1 mutants developed spina bifida at high penetrance (79.3%; Fig. 1C,E,F,I; Table 1), as described previously (Camerer et al., 2010), although a lower frequency of curly tail as the sole phenotype was also observed (13.8%; Fig. 1B,D; Table 1). A dorsally curled tail can result from delayed spinal neural tube closure (Copp, 1985), indicating that more than 90% of Grhl3Cre-Rac1 mutant fetuses exhibit delayed or failed spinal closure. In contrast, 100% of Non-Cre and 97.5% of Grhl3Cre-Con fetuses had normal spinal regions, with only a single case of curly tail observed in the latter group (Table 1).

### Grhl3Cre-Rac1 mutants can display either exencephaly or encephalocele

Three distinct cranial phenotypes are observed in Grhl3Cre-Rac1 mutant fetuses. In our previous study, exencephaly, the developmental forerunner of anencephaly, affected 30% (21/69) of Grhl3Cre-Rac1 embryos at E9.5 and 25% (11/44) at E10.5-13.5 (Rolo et al., 2016). Here, we examined E13.5-17.5 fetuses and found exencephaly in 31% of cases (9/29; Table 1). Hence, exencephaly was first seen at the stage when cranial neural tube closure is usually completed (E9.5) and persisted at a relatively constant rate into later gestation. The defect showed the typical appearance of failed cranial neural tube closure, with an extensive region of everted, open neural folds involving forebrain, midbrain and hindbrain (between white arrows in Fig. 1B,C,H). At E13.5, the exposed neuroepithelium had a voluminous, healthy appearance (Fig. 1B,C), but by E17.5 the exposed tissue had partially collapsed on top of the head and showed haemorrhage (Fig. 1H), indicative of progressive *in utero* degeneration, which ultimately leads to the conversion of exencephaly to anencephaly (Wood and Smith, 1984).

From E13.5 onwards, we also encountered another cranial phenotype resembling parieto-occipital encephalocele. This affected 34.5% (10/29) of Grhl3Cre-Rac1 mutant fetuses, and was present in 50% (10/20) of the non-exencephalic mutants. It did not occur in the control genotypes (Table 1) or concomitant with exencephaly in the same mutant. At E13.5, encephalocele appeared as a distinctly backward-pointing herniation of the more posterior cranial region (Fig. 1D-F). This was in sharp contrast to littermates with exencephaly, which had an extensive ‘mushroom-like’ appearance with the everted neural folds encompassing almost the entire head (Fig. 1B,C). In the majority of cases at E13.5 (Fig. 1D,F), and also later at E17.5 (Fig. 1I), the encephalocele herniation appeared as a smooth projection from the more posterior part of the head (Fig. 1I), with no signs of progressive degeneration, unlike the exencephalic lesions. In a single case, the herniation had a small region of open neural tube at its apex (asterisk in Fig. 1E), suggesting that in a minority of cases the encephalocele might rupture locally.

The remaining 34.5% (10/29) of Grhl3Cre-Rac1 mutants had a normal cranial region in which the neural tube was closed and there was no sign of brain herniation (Fig. 1G). Hence, although almost all mouse mutants lacking Rac1 expression in the surface ectoderm failed in spinal neural tube closure, they had approximately equal frequencies of three distinct cranial phenotypes: exencephaly, occipito-parietal encephalocele and normal cranial region. Importantly, the two abnormalities of cranial development arose in different individuals, at different developmental stages: exencephaly from E9.5 when neurulation finishes in the head and encephalocele from E13.5 onwards, consistent with this being a post-neurulation defect.

### Association between malformations of brain/head and spine in Grhl3Cre -Rac1 mutants

**Fig. 2.**
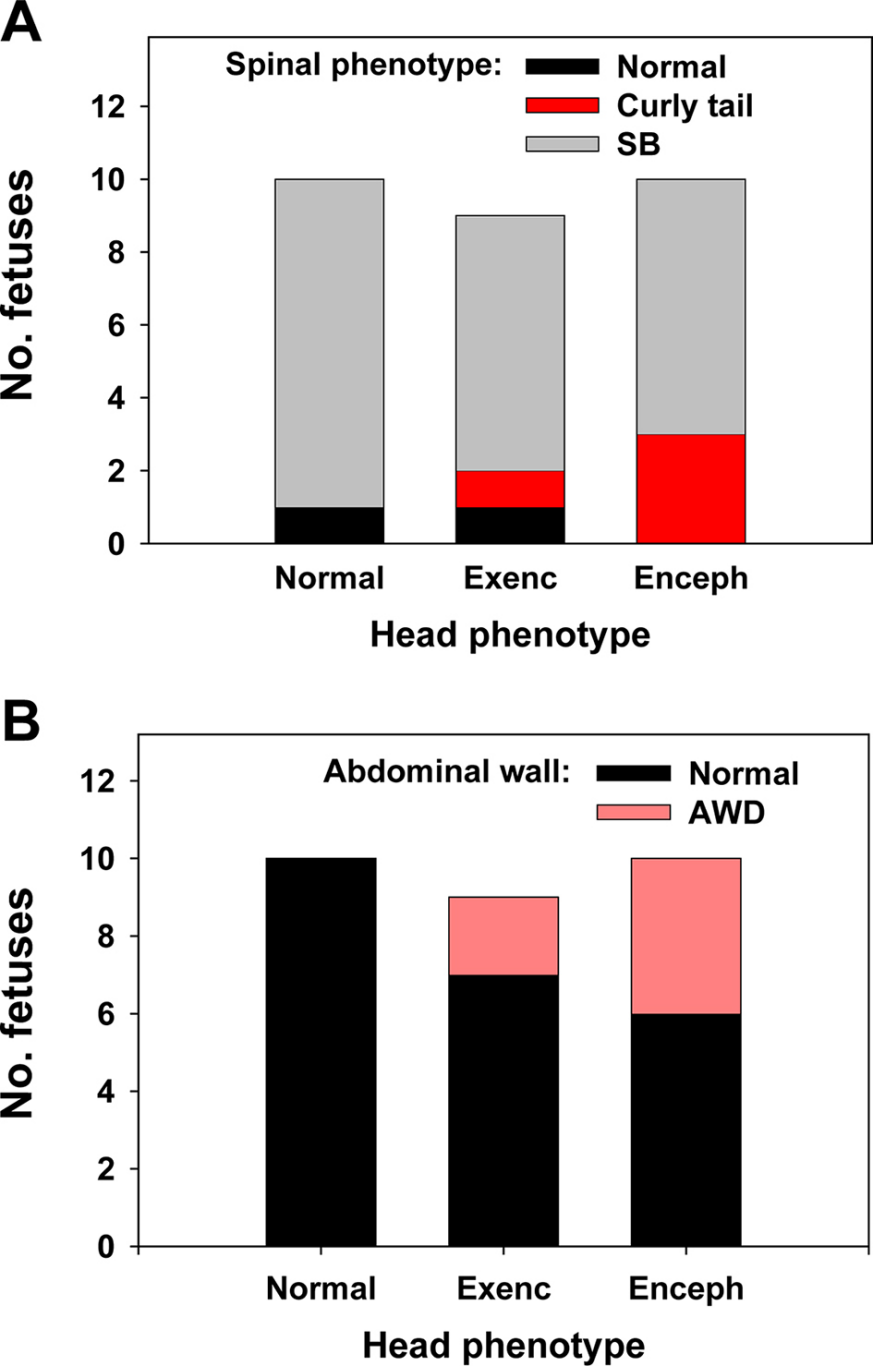
**Association of cranial, spinal and abdominal defects in Grhl3Cre-Rac1 mutants.** (A) Number of Grhl3Cre-Rac1 mutant fetuses (E13.5-E18 pooled; *n*=29) with various combinations of cranial (normal, exencephaly, encephalocele) and spinal (normal, curly tail, spina bifida; SB) phenotypes. The distribution of spinal phenotypes does not differ significantly between the three cranial phenotypes (Chi-squared test; *P*=0.33). (B) Number of Grhl3Cre-Rac1 mutant fetuses (E13.5-E18 pooled; *n*=29) with abdominal wall defect (AWD) among the three cranial phenotypes. AWD was considered present only when the liver was exteriorised, which is an abnormal situation at all stages. AWD was present in 6/19 mutant fetuses with a cranial defect but did not occur in fetuses with normal head (0/10). The frequency of AWD does not differ significantly between the three cranial phenotypes (Chi-squared test; *P*=0.087) but shows a trend towards statistical significance when comparing fetuses with normal versus defective (exencephaly or encephalocele pooled) cranial regions (Fisher's exact test; *P*=0.068).

We asked whether spinal and brain/head phenotypes are statistically associated in Grhl3Cre-Rac1 mutant fetuses. That is, does the presence of a spinal closure defect predict the additional presence of either exencephaly or encephalocele? Spinal defects (spina bifida or curly tail) occurred in fetuses with normal heads (9/10), as well as in those with exencephaly (8/9) and encephalocele (10/10). Interestingly, most mild spinal defects (curly tails) occurred in fetuses with encephalocele (Fig. 2A). Nevertheless, there was no statistically significant association between defects of spine and head. This suggests that, given an overall predisposition to neural tube malformations in Grhl3Cre-Rac1 mutants, the actual risk of a particular defect occurring is independent of other defect types.

### Other developmental defects in Grhl3Cre-Rac1 mutants

In addition to defects of the spine and brain/head, Grhl3Cre-Rac1 mutant fetuses also exhibited abdominal wall defects (AWD). This defect was defined in the study as exteriorisation of the gut and liver outside the abdominal cavity (Fig. 1E,I), which probably represents omphalocele (also called exomphalos). AWD affected 6/29 Grhl3Cre-Rac1 mutant fetuses and was present only in those with a coexisting cranial defect (Fig. 2B), suggesting a possible specific association between AWD and head defects. Nevertheless, the Chi-squared test showed no significant statistical association, probably due to the low frequency of AWD and hence an underpowered analysis. A further defect observed in Grhl3Cre-Rac1 mutant fetuses was occasional microphthalmia (Fig. 1B) or anophthalmia, but this occurred at low frequency and was not analysed further.

### Late-stage morphology of mutant fetuses with encephalocele: skull and brain defects

**Fig. 3.**
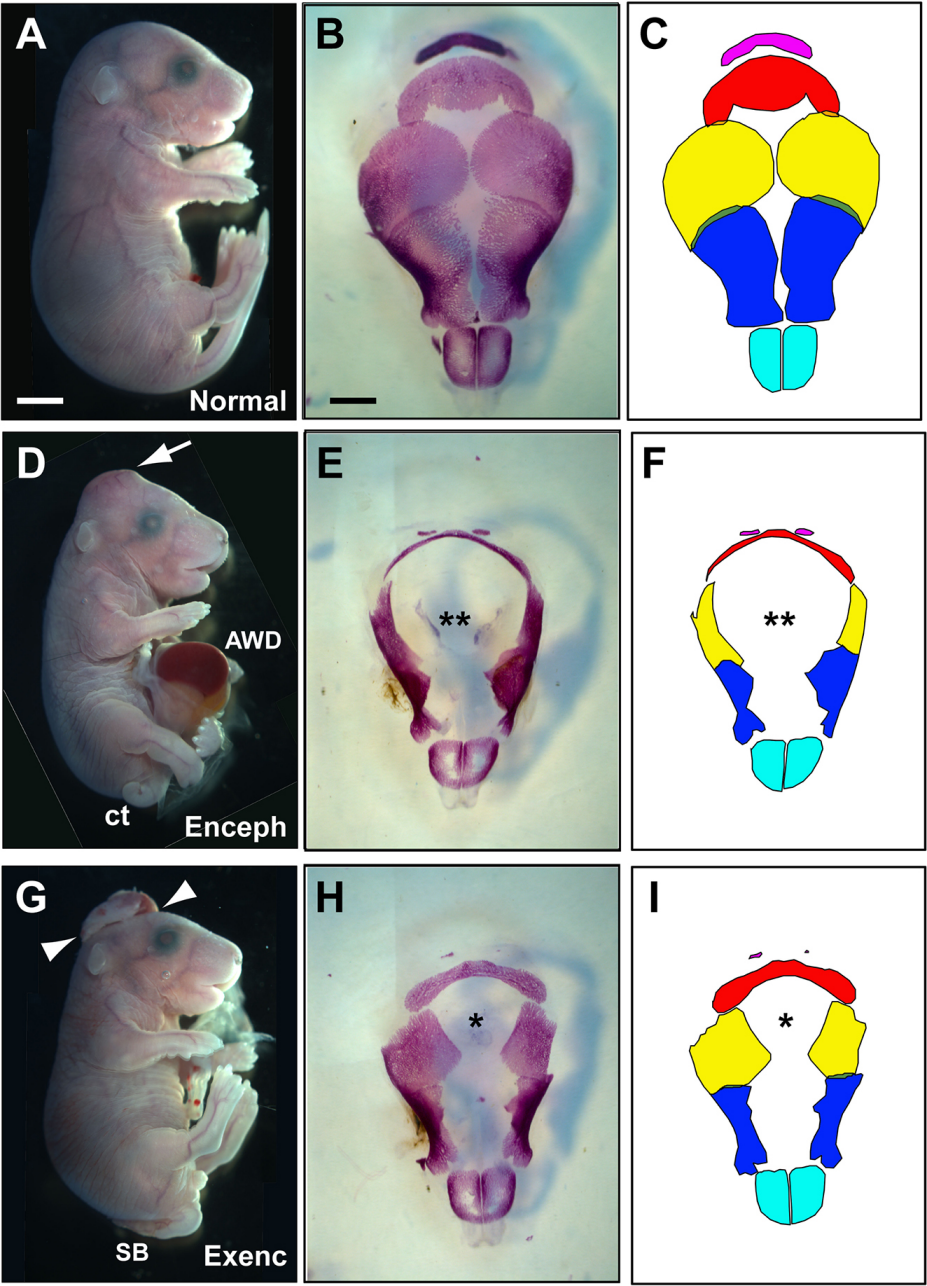
**Grhl3Cre-Rac1 mutants with encephalocele or exencephaly exhibit calvarial skull defects.** Grhl3Cre-Con (A,B,C); and Grhl3Cre-Rac1 (D-I) conditional mutants at E17.5. (A,D,G) Normal head and spinal appearance in a nonmutant fetus (A), whereas the mutant fetuses show occipito-parietal encephalocele (Enceph; arrow in D) and the open NTD exencephaly (Exenc; between arrowheads in G). Both mutants have spinal defects: curly tail (ct in D) and spina bifida (SB in G). The fetus with encephalocele also has an abdominal wall defect (AWD), exposing the internal organs, including liver. (B,E,H) Calvarial skull preparations viewed from the top and (C,F,I) corresponding diagrams to show the identity of the bones: pink, occipital; red, interparietal; yellow, parietal; blue, frontal; cyan, nasal. The bones of the Grhl3Cre-Con fetus are normally formed and meet in the dorsal midline, prefiguring the sagittal suture (B,C). In contrast, a large midline deficit in bone formation occurs in the fetus with encephalocele (**); all bones except the nasals are severely affected in their medial aspects (E,F). Similar, but less severe, defects (*) are present in the fetus with exencephaly (H,I). Analyses were performed on at least three different fetuses of each genotype and phenotype group, with representative specimens shown. Owing to a limited field of microscopic view, multiple photographic images were taken of the samples depicted in panels A,D,G and composite images are displayed. Scale bars: 1 mm (A,D,G), 500 µm (B,E,H).

Skull preparations showed that the calvarial bones of E17.5 Grhl3Cre-Con fetuses were well formed and met at the dorsal midline, prefiguring the sagittal suture (Fig. 3A-C). In contrast, Grhl3Cre-Rac1 mutant fetuses with encephalocele had a large midline deficit in bone formation, where all bones except the nasals were severely affected in their medial aspects (Fig. 3D-F). Similar bone defects were seen in fetuses with exencephaly (Fig. 3G-I) but, strikingly, these were less severe despite the very pronounced exencephalic brain defect (Fig. 1E). Hence, formation of the bony calvarium, a tissue in which Rac1 does not recombine in mutant fetuses, was defective, probably because the persistently open or herniated brain interfered mechanically with moulding of the overlying skull tissue.

**Fig. 4.**
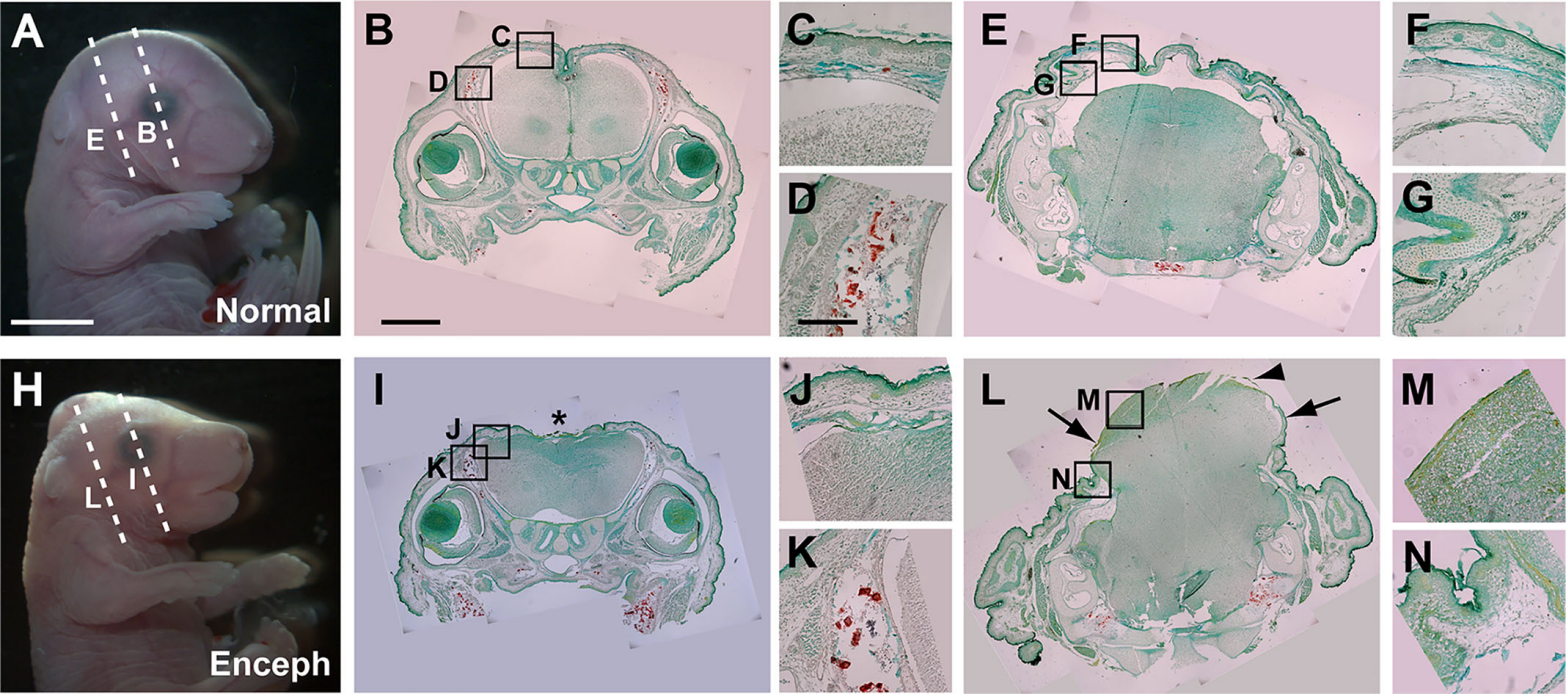
**Structure of late-stage fetal encephalocele in Grhl3Cre-Rac1 mutants.** (A,H) Normal Grhl3Cre-Con fetus (A) and Grhl3Cre-Rac1 conditional mutant with occipito-parietal encephalocele (H) at E17.5. Dashed lines on fetal heads indicate orientation of sections. (B-G,I-N) Low magnification sections through the head are stained with Alizarin Red to reveal mineralised bone (B,E,I,L). Boxed areas are shown at higher magnification in C,D,F,G,J,K,M,N. The section rostral to the encephalocele (I; at level of the eyes) shows a closed neural tube covered by epidermis (J,K), although the dorsal surface is irregular and appears atretic (*) compared with the normal appearance of the Grhl3Cre-Con fetus (B-D). The section taken through the encephalocele (L) shows a massive extrusion of brain tissue from the dorsal surface of the head. Epidermis and subcutaneous tissue are present at the edges of the encephalocele (N) but are absent over most of the brain herniation (M), in contrast to the nonmutant appearance (E-G). However, some residual membranous covering can be observed (arrowhead in L). The brain appears ‘closed’ (i.e. normally neurulated) in the protruded area, although there is clear internal disorganisation. Analyses performed on at least three different fetuses of each genotype and phenotype group, with representative specimens shown. Owing to a limited field of microscopic view, multiple photographic images were taken of the samples depicted in panels B,E,I,L and composite images are displayed. Scale bars: 2 mm (A,H), 500 µm (B,E,I,L), 100 µm (C,D,F,G,J,K,M,N).

Histological sections through the head of Grhl3Cre-Rac1 mutants and control fetuses at E17.5 (Fig. 4A,H) showed that the encephalocele comprised a mass of brain tissue, which lacked the precise internal structure of the control brain (Fig. 4L). For example, no lumen could be discerned in the encephalocele herniation, unlike in the normal brain (compare Fig. 4L and Fig. 4E). The encephalocele was also largely devoid of a tissue covering. Besides the lack of a bony calvarium, the overlying epidermal and mesenchymal tissues were largely absent (Fig. 4L,M), although a thin membrane could be seen covering parts of the dorsal aspect of the encephalocele (arrowhead in Fig. 4L). In contrast, sections at this level of a normal Grhl3Cre-Con fetal head showed both skeletal and soft tissue covering of the brain (Fig. 4E-G). More rostral sections through the head, at a level in front of the encephalocele, showed brain covered by epidermal tissue and mesenchyme in both mutant and control fetuses (Fig. 4B-D,I-K). Bone was absent at this level, as expected from the skull preparations (Fig. 3). Nevertheless, the tissue covering the dorsal aspect of the brain in the fetus with encephalocele was relatively thin and appeared atretic (asterisk in Fig. 4I) compared with the covering in the control fetus (Fig. 4B,C).

In conclusion, late-stage encephalocele in Grhl3Cre-Rac1 fetuses is a massive brain herniation through a large opening in the bony calvarium, with minimal residual tissue covering. Although it demonstrated internal disorganisation, the external surface remained smooth and relatively well preserved, with no obvious degeneration.

### Encephalocele development is preceded by rupture of the surface ectoderm

**Fig. 5.**
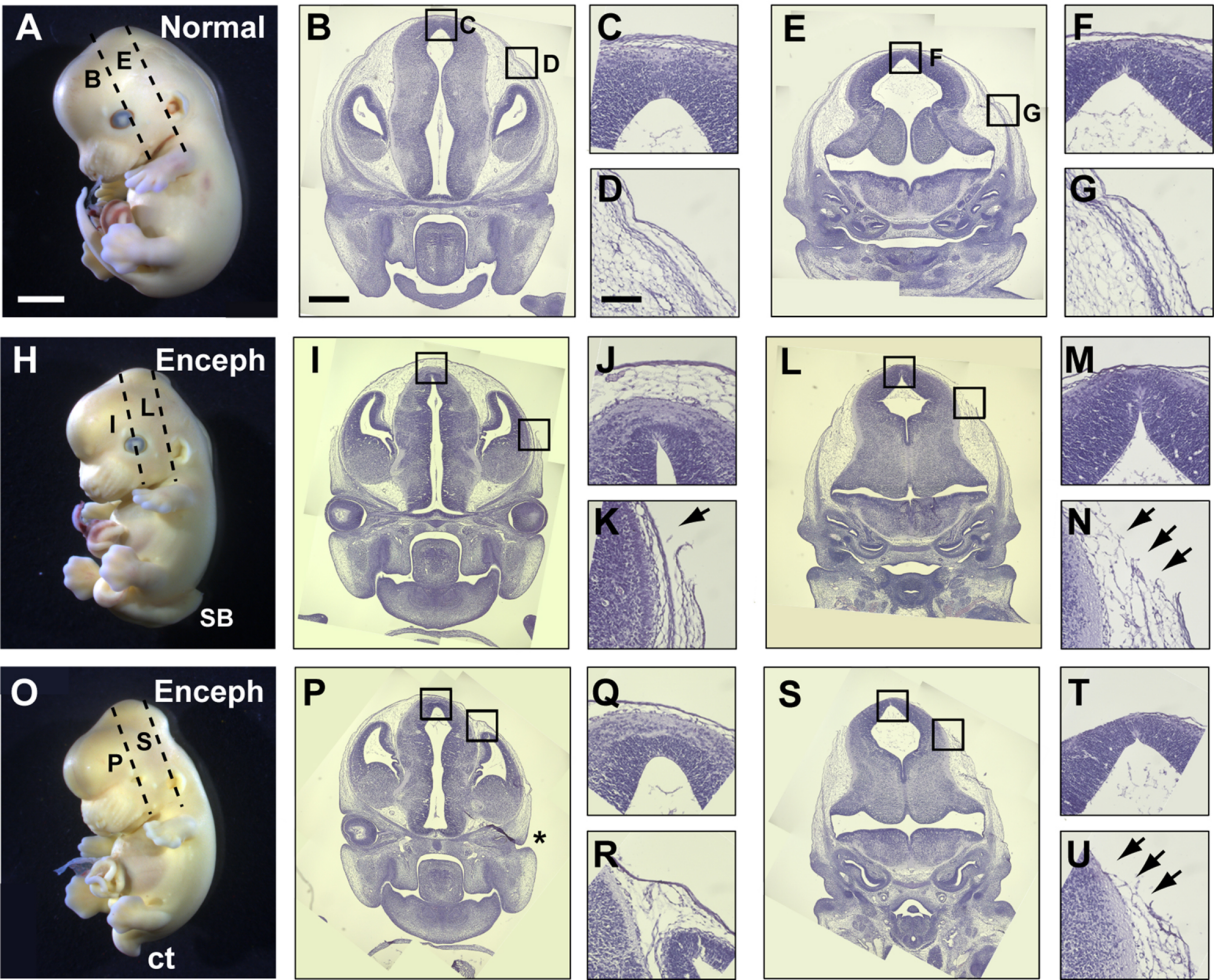
**First appearance of encephalocele in Grhl3Cre-Rac1 mutants.** (A,H,O) Non-Cre control fetus (A) and Grhl3Cre-Rac1 conditional mutants with occipito-parietal encephalocele (Enceph; H,O) at E13.5. Both mutant fetuses have spinal defects: spina bifida (SB in H) and curly tail (ct in O). Left-sided anophthalmia is visible in one mutant fetus (O; see asterisk in P). Dashed lines on fetal heads indicate position and orientation of sections. (B-G,I-N,P-U) H&E-stained sections through the head at levels indicated in A,H,O. Boxed areas in the low magnification sections (B,E,I,L,P,S) are shown at higher magnification in C,D,F,G,J,K,M,N,Q,R,T,U. Disruption of the future epidermis and subcutaneous tissue at the edge of the brain herniation is visible in both mutants (black arrows in K,N,U), whereas the comparable tissues in the nonmutant fetus are intact (D,G). Tissues overlying the dorsal aspects of the brain are intact, although these tissues are thin and attenuated over the incipient encephalocele (J,M,Q,T) compared with the control fetal tissues, which are thicker (C,F). Analyses performed on at least three different fetuses of each genotype and phenotype group, with representative specimens shown. Owing to a limited field of microscopic view, multiple photographic images were taken of the samples depicted in panels B,E,I,L,P,S and composite images are displayed. Scale bars: 500 µm (A,H,O), 200 µm (B,E,I,L,P,S), 40 µm (C,D,F,G,J,K,M,N,Q,R,T,U).

To further address the developmental origins of encephalocele in Grhl3Cre-Rac1 fetuses, we examined sections through the mid- and hind-brain of earlier stage fetuses, at E13.5, when the encephalocele defect first becomes identifiable. At this stage, sections through the incipient encephalocele of two different affected fetuses (Fig. 5H,L-N and Fig. 5O,S-U) showed a closed neural tube, with morphology very similar to that of controls (Fig. 5A,E-G). The neural tube was completely closed and had a well-defined ventricular lumen at all rostro-caudal levels of the brain examined. In contrast, the overlying tissues showed distinct differences between affected and unaffected fetuses: the covering layers were intact and regular in the nonmutant fetus (Fig. 5E-G), but the surface ectoderm and underlying mesenchyme on the dorsolateral aspect of the encephalocele showed breaks and discontinuities in both affected fetuses (arrows in Fig. 5N,U). In contrast, the surface ectoderm overlying the dorsal aspect of the brain was intact in both, although it appeared thinner and more closely adherent to the brain surface than in controls (compare Fig. 5M,T with Fig. 5F). Sections taken rostral to the level of the encephalocele showed dorsolateral surface ectoderm rupture in one affected fetus (arrow in Fig. 5K) but not in the other (Fig. 5R), nor in the normal control (Fig. 5D). These findings confirm that encephalocele arises after apparently normal neural tube closure, as the brain is entirely closed at the stage when the defect first becomes visible. Moreover, the surface ectoderm, which specifically lacks Rac1 in the mutants, showed evidence of rupture at the earliest stages of encephalocele development, suggesting a mechanism of pathogenesis for the brain herniation.

### Assessment of Grhl3Cre-mediated recombination in embryonic tissues

**Fig. 6.**
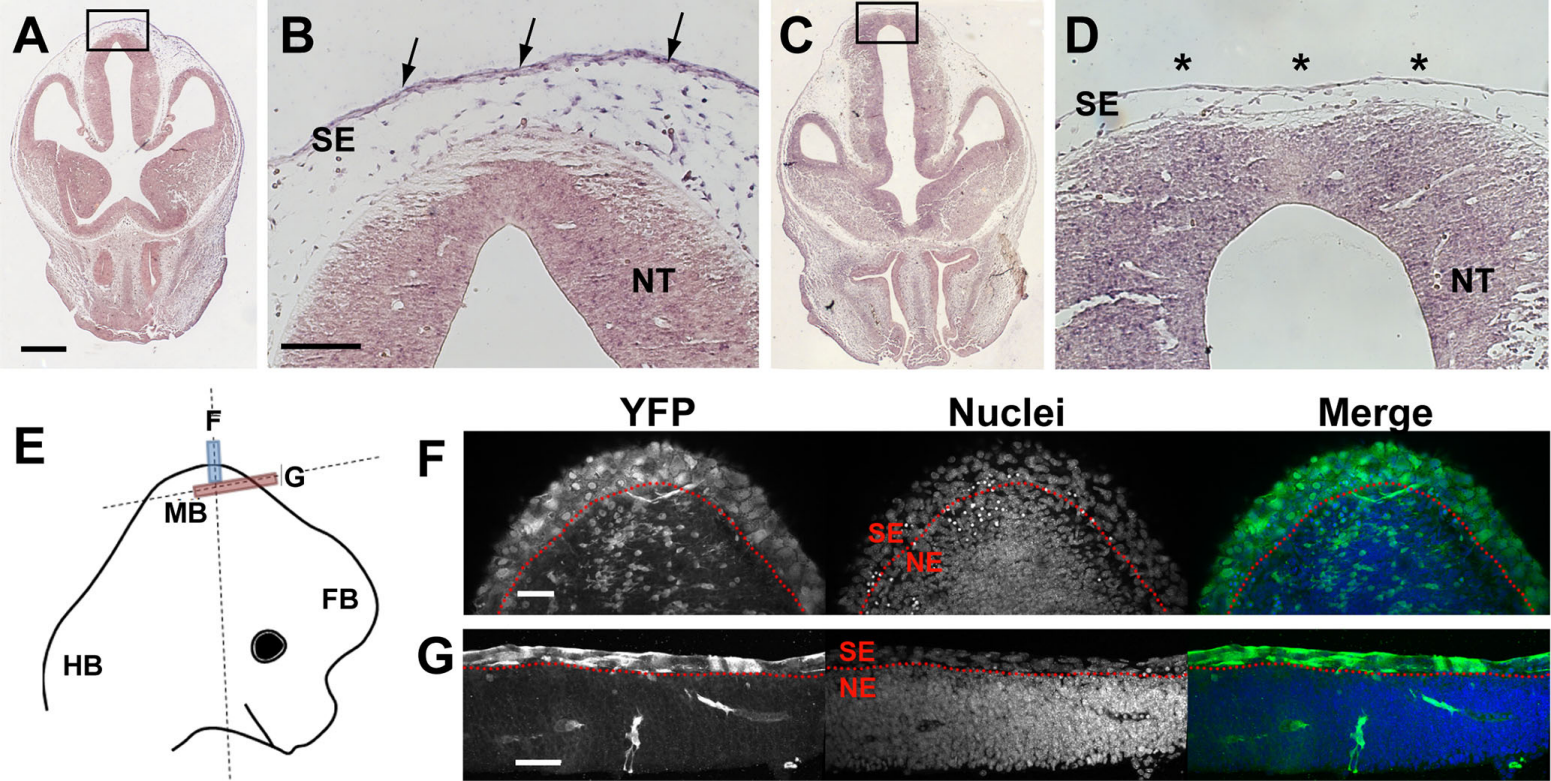
**Knockdown of Rac1 in surface ectoderm by Grhl3Cre-specific recombination.** (A-D) *In situ* hybridisation on coronal sections through the midbrain region at E12.5, using an antisense RNA probe against mouse Rac1, exons 4 and 5. *Rac1* mRNA is expressed (purple staining) in all tissues of the Grhl3Cre-Con embryo (A,B), including the neural tube (NT) and surface ectoderm (SE; arrows in B). Expression is also detected in the NT of the Grhl3Cre-Rac1 conditional mutant (C,D), whereas expression is specifically abolished in the mutant SE (asterisks in D), where Grhl3Cre causes recombination of the floxed Rac1 alleles. Note the thin, attenuated appearance of the SE in the mutant (D), with reduced subepidermal tissue although, at this stage, the mutant NT is still covered by intact SE. (E-G) Schematic of an E12.5 mouse embryo head (E), with dashed lines indicating the planes of section shown in F,G. Coloured boxes show the areas and planes of section. Grhl3Cre-driven recombination of the ROSA26-YFP reporter, as detected by anti-YFP immunofluorescence. YFP expression can be seen in all SE cells (above dotted red line), but only in a few scattered neuroepithelial (NE) cells (below dotted red line). Scale bars: 100 µm (A,C), 50 µm (B,D,F,G).

The defects in Grhl3Cre-Rac1 mutant embryos at E13.5 appeared to localise primarily to the surface ectoderm, rather than to the neural tube. It is important to ask, therefore, whether Grhl3Cre does indeed target Rac1 loss of function primarily in the surface ectoderm, as expected of this Cre driver (Camerer et al., 2010). We examined Rac1 expression by *in situ* hybridisation at E12.5, a day before the encephalocele lesion could be discerned in Grhl3Cre-Rac1 individuals. *Rac1* mRNA was readily detected in both the surface ectoderm and neuroepithelium of nonmutant embryos (Fig. 6A,B) and, although it was also detected in the mutant neural tube, Rac1 expression was specifically abolished in the mutant surface ectoderm (Fig. 6C,D). Moreover, this ectodermal layer appeared thinner, with fewer subectodermal mesenchyme cells, perhaps prefiguring the later rupture of the surface ectoderm.

To detect Grhl3Cre-driven recombination directly at E12.5, we bred mice carrying the Rosa26-EYFP reporter as well as Grhl3Cre. Immunostaining for yellow fluorescent protein (YFP) confirmed that, as at earlier stages (Rolo et al., 2016), recombination occurred in the entire dorsal surface ectoderm, but only in a variable small minority of cells of the neuroepithelium (Fig. 6E-G). We conclude that Grhl3Cre-mediated recombination and Rac1 knockdown occur specifically in the surface ectoderm, prior to the stage of onset of ectodermal rupture and appearance of the encephalocele lesion at E13.5. Hence, this Rac1 loss is probably the cause of subsequent disruption of morphogenesis, leading to occipito-parietal encephalocele.

## DISCUSSION

The availability of animal models has greatly aided our understanding of the embryonic and fetal pathogenesis of congenital defects. For example, mouse and other animal models have contributed to our knowledge of the development of the craniofacial anomaly Treacher–Collins syndrome (Sakai and Trainor, 2009), the enteric nervous system defect Hirschsprung disease (Heanue and Pachnis, 2007) and the congenital heart disorder DiGeorge syndrome (McDonald-McGinn et al., 2015). NTDs comprise the commonest category of congenital nervous system defects, and understanding of their prenatal origins has been enhanced by animal models (Copp et al., 2003). However, information about the developmental basis of encephalocele, a member of the NTD spectrum, has lagged behind because of the lack of a suitable animal model.

### Mouse models of encephalocele

Over 240 different mouse models of NTDs have been reported, mainly displaying exencephaly, spina bifida or both (Harris and Juriloff, 2010). To date, only two convincing mice with genetically determined encephalocele have been described: the *tuft* mouse, which involves a mutation of the *Tet1* gene (Fong et al., 2014, 2016), and the *fog* mutant in which the *Apaf1* gene is mutated (Honarpour et al., 2001). Both display frontal encephalocele together with craniofacial defects and, in *tuft* mice, also lipoma. In humans, fronto-ethmoidal encephalocele is particularly found in Southeast Asia (Tirumandas et al., 2013), but is less common in other geographical locations compared with occipital encephalocele. Moreover, lipoma does not typically accompany any of the varieties of human encephalocele. Our description of the defect in Grhl3Cre-Rac1 mutants is the first report (to our knowledge) of a mouse model of occipito-parietal encephalocele without accompanying craniofacial defects or lipoma. It therefore represents a proof-of-principle study of the origin during brain development of a type of encephalocele (occipital) that is found most commonly in humans.

### Encephalocele as a post-neurulation defect

Although encephalocele is typically classified as an NTD, uncertainty continues over its relationship to neural tube closure. For example, the frontal encephalocele in *tuft* mice was described as resulting from incomplete closure of the anterior neural tube (Fong et al., 2014). On the other hand, some authorities argue that encephalocele is a later-arising defect, resulting from incomplete fusion of skull bones at the midline, creating a gap through which meninges and brain tissue herniate (Tirumandas et al., 2013). The defect in Grhl3Cre-Rac1 mutants is first detected at E13.5, about 4 days after anterior neural tube closure is complete, but before the beginning of skull ossification. In sharp contrast, exencephaly arises in the same mutant litters with an onset at E9.5, the stage of cranial neural tube closure.

We conclude that encephalocele, at least in Grhl3Cre-Rac1 mutants, is neither the result of failure in neural tube closure nor primarily a skull defect. Rather, it develops after neural tube closure is complete as a result of a defect in the surface ectoderm, and the defect is already manifest by the time of skull formation. Despite not being the result of a skull defect, encephalocele in Grhl3Cre-Rac1 mutants is nonetheless associated with severe malformation of calvarial bone formation, perhaps accounting for the later pathogenesis of human encephalocele, in which the brain and/or meninges herniate through a skull defect.

### Does the Grhl3Cre-Rac1 mouse serve as a model for human encephalocele?

Human encephalocele is most often isolated and hence nonsyndromic, although it can be associated with other body system defects, as in the occipital encephalocele of MKS. Although the genetic basis of MKS as a ciliopathy is well established (Logan et al., 2010), the developmental link between compromised ciliary function and encephalocele is unclear. Nonsyndromic encephalocele shares epidemiological features with open NTDs (Rowland et al., 2006) and, as with all NTDs, the available evidence points to multifactorial causation in which the precise combination of genetic and nongenetic predisposing factors varies among affected individuals (Wallingford et al., 2013). In seeking functional genetic variants that predispose to human NTDs (Ross et al., 2017), it has been unclear whether overlapping or different causative factors underlie the different NTDs. The Grhl3Cre-Rac1 mouse model firmly links neural tube closure defects with encephalocele, as both malformation types result from the same gene defect and individual fetuses frequently exhibit both encephalocele and open spina bifida. Moreover, although the single-gene causation of the Grhl3Cre-Rac1 mouse does not model human encephalocele aetiology, it does demonstrate that a single causative factor can produce encephalocele, exencephaly and spina bifida. This provides a renewed impetus to identify predisposing genetic variants that might be relevant across the spectrum of human NTDs.

In humans, the encephalocele is typically covered by skin or at least by a membrane, although this is not universal. For example, in one series (Kotil et al., 2008), only 2/12 large encephaloceles had skin coverage. Loss of skin over encephaloceles has been documented (Berry and Patterson, 1991), whereas re-epithelialisation is also a possibility. Another typical feature of the human condition is its sac-like nature, in which a cavity occurs within the brain/meningeal herniation. At first sight, the Grhl3Cre-Rac1 mouse model seems to depart from both of these typical features of human encephalocele. The brain herniation originates after loss of the surface ectoderm (the future epidermis) and the initial brain projection is solid, not cavitated. However, it should be borne in mind that the Grhl3Cre-Rac1 model represents a very early stage in the emergence of brain herniation. The defect was first observed at E13.5, at the transition from embryo to fetus, equivalent to 8-9 weeks post-conception in human. In contrast, detailed knowledge of human encephalocele has come from studies at late fetal or neonatal stages. Although encephalocele can be detected by first trimester ultrasound (Engels et al., 2016), little detailed structural information can be obtained at such an early stage. Hence, changes during gestation (e.g. cavity formation as cerebrospinal fluid volume increases) may occur between the initial herniation early in brain development and the later appearance of a ‘full-blown’ encephalocele. Longitudinal studies of mouse encephalocele in the postnatal period are not possible because of the typical death of malformed fetuses at birth and their cannibalism by the mother. Hence, further information on possible time-dependent changes in encephalocele during fetal development will require studies of affected humans at earlier developmental stages than has been performed previously.

Although the brain herniation in the Grhl3Cre-Rac1 mouse is not skin-covered, a membranous covering was visible. Correlating with this, we noted a marked difference between encephalocele and exencephaly in the changes that occurred between E13.5 and E17.5. The exencephaly lesions show evidence of *in utero* degeneration, which is known to occur in NTDs after prolonged exposure of neural tissue to amniotic fluid (Stiefel et al., 2007; Wood and Smith, 1984). That the encephalocele lesion did not show similar degeneration in the mouse model is consistent with the possibility of an overlying membrane, albeit thin, providing protection. In open NTDs (exencephaly, open spina bifida), the apical surface of the neuroepithelium is exposed to the amniotic fluid. However, in the Grhl3Cre-Rac1 encephalocele, the basal surface becomes exposed after loss of the surface ectoderm and underlying mesenchyme. The basal surface is normally covered by extracellular matrix, particularly the basement membrane, and it is possible that this persists and provides protection for the exposed brain tissue. It could also provide a substrate for subsequent re-epithelialisation. It will be interesting to test whether early stage human encephaloceles similarly have basement membrane material on their outer surfaces.

### Developmental basis of the requirement for Rac1 in surface ectoderm

Rac1 is required for many cellular processes, including maintenance of cell proliferation, integrity of epithelial cell junctions and cytoskeletal events in cell shape change and motility (Etienne-Manneville and Hall, 2002). Constitutive inactivation of Rac1 is lethal at an early embryonic stage before neurulation begins (Sugihara et al., 1998) and so Rac1 function *in vivo* has been investigated by conditional gene targeting, as in the present study. For example, tissue-specific depletion of Rac1 in the early embryo causes defective cell migration, both of the anterior visceral endoderm (Migeotte et al., 2010), which is required for head induction, and of the mesoderm during subsequent gastrulation (Migeotte et al., 2011). Neural crest migration and differentiation are defective in the absence of Rac1 (Fuchs et al., 2009; Shoval and Kalcheim, 2012; Thomas et al., 2010). Both canonical and noncanonical Wnt signalling require Rac1 for full function (Boczonadi et al., 2014; Tan et al., 2008). Most important for the present study is the finding that inactivation of Rac1 in adult skin leads epidermal stem cells to exit from the cell cycle and undergo differentiation (Benitah et al., 2005). Hence, loss of Rac1 in the surface ectoderm overlying the brain in our study may limit tissue expansion and hence predispose to loss of tissue integrity, enabling brain herniation.

Brain herniation and defective closure of the neural tube and abdominal wall all coexist in the mouse model, prompting the following question: Does the same Rac1-related pathogenic mechanism apply in each case? We found evidence of surface ectoderm rupture, preceding brain herniation in fetuses with encephalocele. This is in sharp contrast to open spina bifida in Grhl3Cre-Rac1 mutants, in which defective neural tube closure results from a lack of cellular protrusions on surface ectoderm cells. These protrusions are required to enable the tips of the neural folds to fuse and complete closure (Rolo et al., 2016). A similar cell protrusion-based mechanism may underlie the failed cranial neural tube closure that leads to exencephaly, although this requires further detailed study. It is unknown how body wall closure fails in some fetuses lacking Rac1 in the surface ectoderm. Hence, the available evidence suggests that Rac1 is required in surface ectoderm cells for at least two distinct functions: to promote cellular protrusive activity during neurulation and to maintain tissue integrity at later stages.

### Are ‘open’ NTDs and encephalocele aetiologically distinct?

Encephalocele was recently identified as part of a ‘cluster’ of NTDs in the pregnancies of HIV-positive Botswanan women exposed, from the time of conception, to the drug dolutegravir as part of triple antiretroviral therapy (Zash et al., 2018; Zash et al., 2019). It is important to determine whether this cluster of NTDs represents a causal link with dolutegravir or is a sporadic association. The former would prompt a re-evaluation of the use of dolutegravir in early pregnancy, despite its high efficacy in preventing vertical HIV transmission (Vitoria et al., 2018), whereas a chance association would not preclude its use in pregnancy. NTDs in the cluster comprised two cases of myelomeningocele (open spina bifida) and one case each of anencephaly, encephalocele and iniencephaly. This broad range of NTDs could argue against causation by a single agent such as dolutegravir. However, our finding that ‘open’ NTDs (spina bifida, exencephaly) can coexist with the post-neurulation defect encephalocele demonstrates that a single causative agent can indeed cause most of these NTDs. This increases the chance that the NTD cluster represents a causal association with dolutegravir exposure. Further studies are needed to fully evaluate the safety of dolutegravir use in early human pregnancy.

### Conclusion

This study describes the first mouse genetic model with brain herniation resembling human occipito-parietal encephalocele. The findings support a post-neurulation origin for encephalocele and demonstrate that brain herniation and failure of brain/spine neural tube closure can all occur as possible developmental outcomes of an identical genetic defect. Skull bone formation follows herniation, and a significant calvarial bone defect occurs over the herniated brain, as in human encephalocele. The mouse model involves conditional deletion of Rac1 expression, mainly in the embryonic and fetal surface ectoderm, and this leads to rupture of surface ectoderm, preceding and probably causing brain herniation. Future challenges include a focus on Rac1 and the several signalling pathways in which it functions as a possible site of genomic and/or epigenomic changes that may predispose to nonsyndromic encephalocele. Moreover, the mouse model provides a tractable system for experimental analysis of early encephalocele development, including interaction with other predisposing genetic variants and with nongenetic factors such as altered folate status.

## MATERIALS AND METHODS

### Mouse procedures and experimental design

Mouse studies were conducted under the auspices of the UK Animals (Scientific Procedures) Act 1986 as described in Project Licence 70-7469, which was scrutinised and approved by the Animal Welfare and Ethical Review Body of University College London. Mice were housed under standard conditions with environmental enrichment. Strains were *Grhl3^Cre/+^* (Camerer et al., 2010), *Rac1^f/f^* (Glogauer et al., 2003) and *ROSA26-EYFP* (Srinivas et al., 2001), all on a C57BL/6 background. Matings were *Grhl3^Cre/+^; Rac1^f/+^* × *Rac1^f/f^*, or *Grhl3^Cre/+^;* *Rac1^−/+^* × *Rac1^f/f^* (Rolo et al., 2016). Fetuses were dissected in Dulbecco's modified Eagle's medium (DMEM; Invitrogen) containing 10% fetal bovine serum (Sigma-Aldrich), and rinsed in phosphate buffered saline (PBS) prior to fixation. Genotyping was by PCR of yolk sac DNA, as described (Rolo et al., 2016). Experiments were conducted according to the ARRIVE guidelines (www.nc3rs.org.uk); for example, analyses were performed blind to genotype, which was obtained after data collection had been completed. All fetuses for which unambiguous genotype and phenotype assessments could be obtained were included in the analysis.

### Histology and skull preparations

Fetuses were fixed over several days in Bouin's solution (Sigma-Aldrich) or in 4% paraformaldehyde in PBS, dehydrated in an ethanol series and embedded in paraffin wax. Sections (5 µm thickness) were stained with Harris' haematoxylin solution and 2% Eosin Y, or Alizarin Red and Fast Green (all Sigma-Aldrich). Images were captured on an Axiophot2 upright microscope. For skull preparations, fetal heads were skinned and stained with Alizarin Red (0.15%) in 1% KOH and cleared with 1% KOH in 20% glycerol (Peskett et al., 2017).

### mRNA *in situ* hybridisation

*In situ* hybridisation was performed on 5 µm thick paraffin sections using a digoxigenin-labelled antisense RNA probe designed to detect the exons deleted in the *Rac1* conditional mutant (Rolo et al., 2016). Images were captured on an Axiophot2 upright microscope.

### Immunofluorescence

Embryos (E12.5) were fixed for 24 h in 4% paraformaldehyde in PBS, pH 7.4, at 4°C. Immunofluorescence for YFP was performed on 12 µm thick cryosections of gelatine-embedded embryos [7.5% gelatine (Sigma-Aldrich) in 15% sucrose] using an anti-GFP (green fluorescent protein) rabbit polyclonal Alexa Fluor 488-conjugated antibody (Life Technologies A21311) at 1:400 dilution. Anti-GFP crossreacts with YFP. Nuclei were labelled with TO-PRO-3 (Thermo Fisher Scientific). Images were captured on an LSM880 Examiner confocal system (Carl Zeiss, UK) as previously reported (Galea et al., 2017); linear adjustments were made using Fiji software.

### Sample size and statistical analysis

Chi-squared and Fisher's exact tests (Sigmastat, v3.5, Systat Software) were used for comparison of phenotype frequencies (Table 1; Fig. 2). A power calculation was performed with the hypothesis that the Non-Cre and Cre-Con genotypes would each show only rare cranial defects (90% normal, 5% exencephaly, 5% encephalocele), whereas the Grhl3-Rac1 group would exhibit approximately equal frequencies of these three outcomes (33% normal, 33% exencephaly, 33% encephalocele). For power equal to 0.9 and *P*=0.05, this generates a sample size of 44, requiring 132 fetal samples across the three genotype groups. Hence, we collected slightly in excess of this number (*n*=142 total fetuses) for final analysis.

## Supplementary Material

Supplementary information
