# Supplementary material for: Novel mouse model of encephalocele: post-neurulation origin and relationship to open neural tube defects
Source: Dis Model Mech. 2019 Nov 14;12(11):dmm040683. doi: 10.1242/dmm.040683 (PMC6899037; doi:10.1242/dmm.040683)
Supplement: Supplementary information [file dmm-12-040683-s1.pdf]

Supplementary data set

Click here to Download Data S1

Disease Models & Mechanisms • Supplementary information
